# Distinctive genotypes in infants with T‐cell acute lymphoblastic leukaemia

**DOI:** 10.1111/bjh.13613

**Published:** 2015-07-24

**Authors:** Marcela B. Mansur, Frederik W. van Delft, Susan M. Colman, Caroline L. Furness, Jane Gibson, Mariana Emerenciano, Helena Kempski, Emmanuelle Clappier, Hélène Cave, Jean Soulier, Maria S. Pombo‐de‐Oliveira, Mel Greaves, Anthony M. Ford

**Affiliations:** ^1^ Centre for Evolution and CancerThe Institute of Cancer ResearchLondonUK; ^2^ Paediatric Haematology‐Oncology Program, Research CentreInstituto Nacional de CâncerRio de JaneiroBrazil; ^3^ Centre for Biological SciencesUniversity of SouthamptonSouthamptonUK; ^4^ Paediatric Malignancy Cytogenetics UnitInstitute of Child Health & Great Ormond Street HospitalLondonUK; ^5^ Department of GeneticsRobert Debré HospitalAPHPParisFrance; ^6^ Haematology LaboratorySaint‐Louis Louis HospitalAPHPParisFrance

**Keywords:** infant, T‐cell acute lymphoblastic leukaemia, genomic profile, CNAs and *in utero* origin

## Abstract

Infant T‐cell acute lymphoblastic leukaemia (iT‐ALL) is a very rare and poorly defined entity with a poor prognosis. We assembled a unique series of 13 infants with T‐ALL, which allowed us to identify genotypic abnormalities and to investigate prenatal origins. Matched samples (diagnosis/remission) were analysed by single nucleotide polymorphism‐array to identify genomic losses and gains. In three cases, we identified a recurrent somatic deletion on chromosome 3. These losses result in the complete deletion of *MLF1* and have not previously been described in T‐ALL. We observed two cases with an 11p13 deletion (*LMO2*‐related), one of which also harboured a deletion of *RB1*. Another case presented a large 11q14·1‐11q23·2 deletion that included *ATM* and only five patients (38%) showed deletions of *CDKN2A/B*. Four cases showed *NOTCH1* mutations; in one case *FBXW7* was the sole mutation and three cases showed alterations in *PTEN*. *KMT2A* rearrangements (*KMT2A‐r*) were detected in three out of 13 cases. For three patients, mutations and copy number alterations (including deletion of *PTEN*) could be backtracked to birth using neonatal blood spot DNA, demonstrating an *in utero* origin. Overall, our data indicates that iT‐ALL has a diverse but distinctive profile of genotypic abnormalities when compared to T‐ALL in older children and adults.

Acute lymphoblastic leukaemia (ALL) in children is a diverse cancer characterized by associations between age at presentation, leukaemic subtype and recurrent genetic alterations (Pui *et al*, 2004). ALL in infants is a rare subset often associated with *KMT2A* (also known as *MLL*) rearrangements (*KMT2A‐r*), a high leucocyte count at diagnosis, an immature or pro‐B‐cell lineage immunophenotype (CD10^−^) and a prenatal origin *in utero* (Ford *et al*, 1993; Biondi *et al*, 2000). T‐ALL is prevalent in older children. Though there is molecular evidence that it can originate *in utero* (Ford *et al*, 1997; Eguchi‐Ishimae *et al*, 2008), it is a very rare disease in infants (Biondi *et al*, 2000; Emerenciano *et al*, 2013). In contrast to infant pro‐B‐lineage ALL with *KMT2A‐AFF1* fusion, in which an *in utero* origin has been clearly demonstrated (Ford *et al*, 1993; Gale *et al*, 1997), the developmental timing for T‐ALL is poorly defined.

In a previous study of T‐ALL, we evaluated fifteen cases in early childhood (age ≤24 months) for mutations that are prevalent in infant ALL (pro‐B) or T‐ALL; *NOTCH1* mutations, although found less frequently than described for older T‐ALL paediatric cases, were the most frequent alterations among these younger patients, followed by the *KMT2A‐r* (Emerenciano *et al*, 2006; Mansur *et al*, 2010).

The availability of a unique series of 13 infant T‐ALL cases (iT‐ALL, ≤12 months) along with 12 remission samples allowed us to determine the molecular profile of iT‐ALL [copy number alterations (CNAs)/gains and losses (loss of heterozygosity, LOH)] using high‐density Genome‐Wide single nucleotide polymorphism (SNP) array accompanied by next generation sequencing (NGS). We sought to investigate the possible prenatal onset of genetic abnormalities in iT‐ALL using a ‘backtracking’ approach with neonatal blood spots (Guthrie cards).

## Materials and methods

### Patient samples

Seven Brazilian (BR1‐BR7), one English (UK1) and five French (FR1‐FR5) iT‐ALLs were included in this study (Supporting Information). Material from diagnostic bone marrow (BM) and/or peripheral blood (PB) was available from all patients and remission samples (non‐leukaemic) were collected for all but one patient (BR4, who did not achieve remission). Guthrie cards were obtained from four patients for use in our backtracking approach to trace prenatally acquired mutations.

### Leukaemia characterization

In all cases, diagnosis of leukaemia was established by the morphology of lymphoid cells and immunophenotyping by flow cytometry using a previously established panel of monoclonal antibodies (Mansur *et al*, 2009). The immunological classification of T‐ALL was performed according to the European Group for the Immunological Characterization of Leukaemias (EGIL) criteria (Bene *et al*, 1995).

### T‐ALL molecular screening

Diagnostic DNA samples from all iT‐ALL cases were analysed for the following gene abnormalities: *NOTCH1*,* FBXW7*,* PTEN*,* IL7R*,* KRAS*,* NRAS*,* STIL‐TAL1 + , TLX3 + * and *KMT2A‐r* (Weng *et al*, 2004; Mansur *et al*, 2009, 2012; Zenatti *et al*, 2011; Emerenciano *et al*, 2013). T‐cell receptor gene rearrangements (*TR‐r*; gamma/*TRG*, delta/*TRD* and beta/*TRB*) were assessed using conditions recommended by the BIOMED‐2 Consortium (van Dongen *et al*, 2003; Langerak *et al*, 2012). Clonality was assessed by GeneScan^®^ profiling (Applied Biosystems^®^, Waltham, MA, USA) followed by cloning of the products and Sanger sequencing. Sequences were analysed using the Ig BLAST (www.ncbi.nlm.nih.gov/igblast/) and the ImMunoGeneTics database (www.imgt.org).

### Molecular analyses

Fluorescence *in situ* hybridization (FISH), CNA analyses, NGS and backtracking of neonatal blood spots were all performed as described in Data S1.

## Results

### Characterization of infant cases

Thirteen iT‐ALL cases were investigated. The median age at diagnosis was 9 months, there was no predominance of gender, and a high leucocyte count (≥50 × 10^9^/L) was observed in 12 out of 13 cases (Table SI). Immunophenotype analyses performed on all 13 diagnostic cases revealed that six patients presented T‐IV profile, five cases T‐III and for the other two cases, one presented T‐I and the other a T‐II profile. The T‐I profile case (BR4) also expressed two classical myeloid markers CD13 and CD33 which, according to previously published criteria (Coustan‐Smith *et al*, 2009), suggests an Early T‐cell Progenitor (ETP) leukaemia.

### Molecular analysis

The main results from the targeted molecular analyses carried out on the iT‐ALL samples are shown in Table 1.

**Table 1:** Clinical‐molecular characterization of infant T‐cell acute lymphoblastic leukaemia cases

Patient ID	Age (months)	Gender	EGIL	TR‐r	NOTCH1	FBXW7	PTEN	IL7R	KRAS/NRAS	STIL‐TAL1	KMT2A‐r	Outcome
*BR1*	12	Male	T‐IV	*TRG & D*	HD Mut	Mut	WT	WT	WT	Neg	Neg	Deceased
*BR2*	8	Male	T‐IV	*TRG, D & B*	WT	WT	WT	WT	WT	Neg	*MLLT1*	Deceased
*BR3*	6	Female	T‐IV	*TRG, D & B*	WT	WT	WT	WT	WT	Neg	Neg	Deceased
*BR4* a	7	Female	T‐I	No Rear	WT	WT	WT	WT	WT	Neg	Neg	Deceased
*BR5* a	11	Male	T‐II	*TRG, D & B*	WT	WT	WT	WT	WT	Neg	Neg	Deceased
*BR6* a	7	Female	T‐III	*TRG, D & B*	PEST Mut	WT	WT/del	WT	WT	Neg	*MLLT1*	Alive/CCR
*BR7* a	8	Male	T‐III	*TRD*	WT	WT	WT	WT	WT	Neg	Neg	Deceased
*UK1*	9	Male	T‐III	*TRG*	WT	WT	WT	WT	WT	Neg	*MLLT4*	Deceased
*FR1*	9	Female	T‐III	*TRG & D*	WT	WT	Mut	WT	WT	Neg	Neg	Alive/CCR
*FR2*	11	Female	T‐IV	*TRG, D & B*	WT	WT	WT	WT	WT	Neg	Neg	Deceased
*FR3*	12	Male	T‐III	*TRG & D*	HD/PEST Mut	WT	Mut	WT	WT	Neg	Neg	Deceased
*FR4*	11	Female	T‐IV	*TRG, D & B*	HD Mut	WT	WT	WT	WT	Neg	Neg	Deceased
*FR5*	9	Female	T‐IV	*TRG & D*	WT	Mut	WT	WT	WT	Neg	Neg	Alive/CCR

ID, identification; EGIL, European Group for the Immunological Characterization of Leukaemias classification; *TR‐r*, T‐cell receptor rearrangements; Mut, mutated; WT, wild type; HD, Heterodimerization Domain; PEST, polypeptide enriched in proline, glutamic acid, serine and threonine domain; Neg, negative; Pos, positive; *KMT2A‐r, KMT2A* rearranged, *KMT2A* (also known as *MLL*); *MLLT1* (also known as *ENL*); *MLLT4* (also known as *AF6*); CCR, complete continuous remission. PEST and TAD (transactivation domain) are both designated as PEST domain only.

a Patients with available Guthrie cards (GC or neonatal blood spots).

The 13 diagnostic infant samples were screened for the known recurrent mutations in T‐ALL, including *NOTCH1*,* FBXW7*,* PTEN* and *IL7R* (detailed mutation data is shown in Table SII), as well as *KRAS*,* NRAS* mutations, *STIL‐TAL1* fusion and the presence of *TLX3*. Results showed four cases were mutated for *NOTCH1*, two being mutated in the HD domain only (BR1 and FR4), one in the PEST only (BR6) and one (FR3) with mutations in both HD and PEST. Patient BR1 presented a combined *NOTCH1/FBXW7* mutation. One case (FR5) presented a sole *FBXW7* mutation. Three cases presented *PTEN* alterations (FR1 and FR3 as mutations and BR6 as CNA/deletion) and all patients were *IL7R*,* KRAS* and *NRAS* wild type (WT). *KMT2A‐r* was confirmed in three cases (two *KMT2A‐MLLT1* and one *KMT2A‐MLLT4*) and, distinct from childhood T‐ALL, we observed no infants with either *STIL‐TAL1*+ or *TLX3*+. *TR‐r* analyses were performed and all but one case (BR4) showed clonal rearrangements (Table 1).

### SNP‐array and FISH data

All diagnostic samples were analysed by SNP‐array to identify genomic losses (LOH) and gains (Table SIII), although one sample (BR1) had a low contrast quality control (CQC, Table SIV). The DNA from this sample was extracted from diagnostic BM slides, from which we were able to identify two alterations: *TR* monoclonal rearrangements and *CDKN2A* homozygous deletion. Both results were confirmed using polymerase chain reaction (PCR) and quantitative PCR (Q‐PCR) approaches.

**Figure 1:**
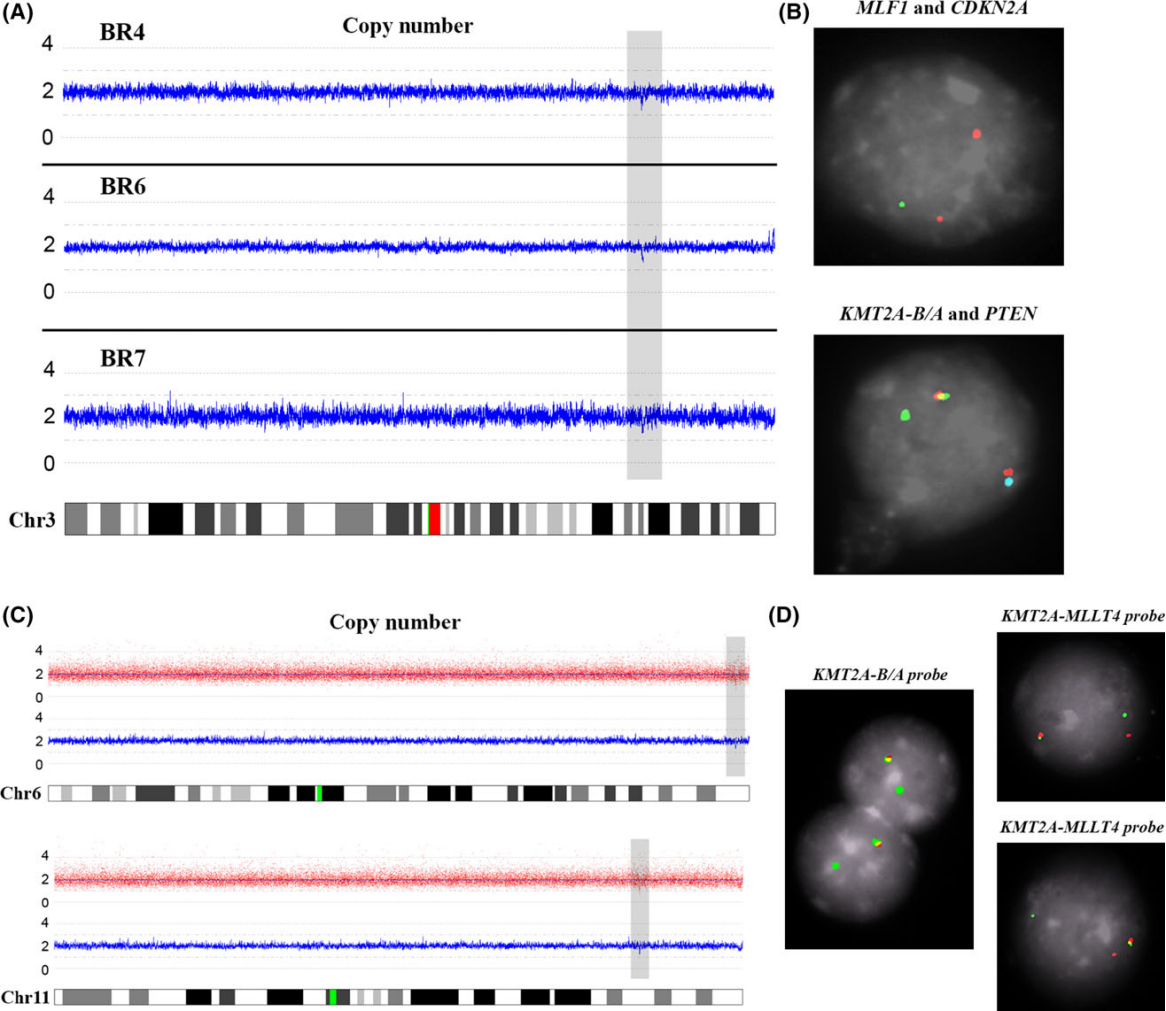
Chromosome aberrations in infant T‐cell acute lymphoblastic leukaemia. (A) Copy number analysis of chromosome 3. The deleted region of chromosome 3 (*MLF1* included) is highlighted in grey for three of our patients (BR4, BR5 and BR6); (B) *MLF1* (spectrum green) and *CDKN2A* (spectrum orange) in‐house probes confirming *MLF1* deletion in patient BR6 & *KMT2A* Break Apart (spectrum orange/spectrum green) and *PTEN* (biotin‐Cy5/turquoise) probes showing *KMT2A‐r* and single copy of *PTEN* in patient BR6. (C) and (D) SNP‐array and FISH analysis of patient UK1. (C) The grey boxes highlight the deleted areas in chromosomes 6 and 11, respectively; (D) FISH using LSI *MLL* (*KMT2A*) Dual Colour, Break Apart Rearrangement probe (spectrum orange/spectrum green) showing the *KMT2A* deletion (one orange signal missing); and in‐house probes, both designed in non‐deleted areas of *MLLT4*‐Cy3 and *KMT2A*‐spectrum green, confirming the *KMT2A‐r* between these two genes.

Among the CNAs identified by SNP‐array, we highlight genes considered as ‘drivers’ of the leukaemic process i.e. those genes already causally implicated in the process of oncogenesis (Table SIII). A recurrent 3q25·32 deletion was observed in three out of 13 cases (BR4, BR6 and BR7; Fig 1A) that encompassed *MLF1* (myeloid leukaemia factor 1), a negative regulator of cell cycle progression which functions upstream of the tumour suppressor *TP53* (Yoneda‐Kato *et al*, 2005). For these 3 cases the deletion range varied between 528,000 bp and 610,000 bp but each deletion encompassed the entire *MLF1* gene. *MLF1* deletion was confirmed by FISH on case BR6, using a combination of *MLF1* and *CDKN2A* in‐house probes (Fig 1B). For the same patient, we also used FISH to confirm both *KMT2A‐r* and *PTEN* deletion (Fig 1B). Using a SP6·0‐array approach, we could not detect deletion of *MLF1* in over 90 European and Brazilian cases of childhood and adolescent T‐ALL (unpublished data).

SNP‐array analyses also revealed one case (UK1) to harbour small deletions in *KMT2A* (11q23) and *MLLT4* genes (6q27) (Fig 1C) and, because *MLLT4* is recognized as a classical *KMT2A* translocation partner gene, we used FISH to search for a potential *KMT2A‐r*. Consequently, we first detected a *KMT2A* deletion using the LSI *MLL (KMT2A)* Dual Colour, Break Apart probe (Fig 1D) and then, using in‐house FISH probes for both *KMT2A* and *MLLT4*, we confirmed the occurrence of *KMT2A‐r* (Fig 1D).

In common with non‐infant paediatric T‐ALL, two of our infant cases revealed an 11p13del (BR3 and BR5; Fig S1), a deletion first described in T‐ALL at a frequency of 4% that also involves region 11p12 (Van Vlierberghe *et al*, 2006). Two other studies on childhood T‐ALL have identified the same deletion (Mullighan *et al*, 2008; Szczepanski *et al*, 2011). A large 11q14‐q23 deletion (Fig S1) including ‘driver’ genes (*ATM*,* EED*) was observed in one case (FR4) while in another, (BR5), we observed a 13q14·2 deletion that involved the *RB1* gene (Fig S2).

**Figure 2:**
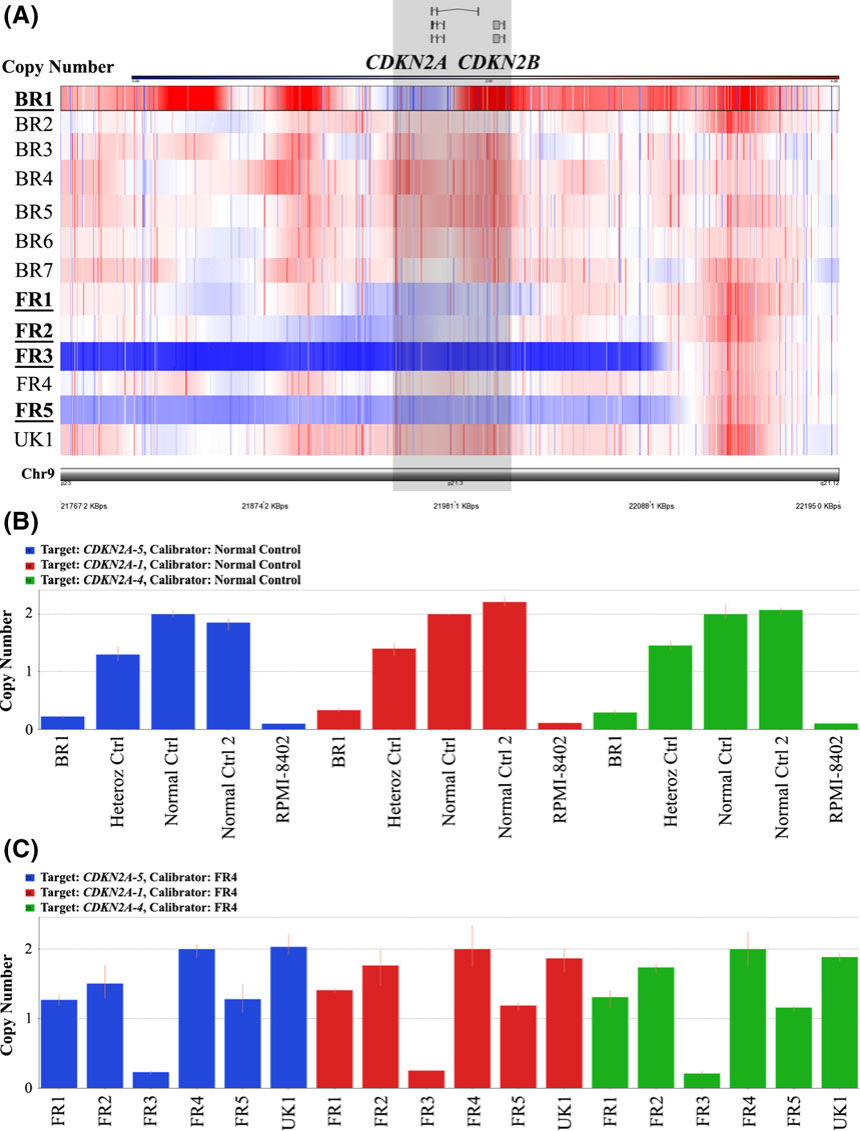
Genomic copy number analysis of *CDKN2A*. (A) SNP‐array analysis of 9p21·3 for the 13 infant T‐cell acute lymphoblastic leukaemia patients, highlighting cases harbouring *CDKN2A/B* deletions (BR1, FR1, FR2, FR3 and FR5), the deleted areas are shown in blue; and (B) and (C) Q‐PCR copy number analysis with three different assays for *CDKN2A*. (B) The charts show patient BR1 as homozygous deleted, also included in the analysis are one heterozygous control, two normal controls and RPMI‐8402 (*CDKN2A* homozygous deleted). (C) Q‐PCR copy number data for patient UK1 and all French cases showing *CDKN2A* heterozygous deletion for patients FR1, FR2 and FR5 and homozygous deletion for FR3, FR4 and UK1 are both *CDKN2A* wild‐type (WT).

Further analysis of our iT‐ALL cohort revealed a lower frequency of *CDKN2A* deletions than found in paediatric T‐ALL. These deletions occur in 70% of T‐ALL (Mullighan *et al*, 2008), but in our study only 38% of iT‐ALL cases harboured this deletion (Fig 2).

### Copy number assays – Q‐PCR and promoter methylation status of CDKN2A

In order to confirm the *CDKN2A* SNP‐array data (Fig 2A) on the five patient samples showing 9p21·3 deletions, we performed real‐time Q‐PCR assays on all 13 diagnostic samples using three different copy number probes located across the gene. In Fig 2B we highlight in particular the SNP analysis for case BR1, for which the array data presented lower CQC compared with the other 12 cases. In this experiment, we used two normal control DNAs (2 *CDKN2A* copies), one heterozygous deleted control (1 *CDKN2A* copy) and DNA from the cell line RPMI‐8402 as a homozygous deleted control, (0 *CDKN2A* copies). Evaluation of the *CDKN2A* status in the other iT‐ALL cases is shown in Fig 2C and Fig S3.

As the majority of our cases did not present a visible *CDKN2A* gene deletion, we explored a potential alternative pathway for *CDKN2A* inactivation, i.e. occurrence of *CDKN2A* promoter methylation. Accordingly, we performed methylation‐specific PCR on all 13 iT‐ALL patient samples and on two control cell lines: RAJI and HL60 (Supporting Information). Conventional Sanger sequencing of the PCR amplicons established the correct genomic location within the *CDKN2A* exon 1 CpG island and confirmed bisulfite modification of the unmethylated cytosine to uracil. As expected, the DNA from the RAJI cell line produced a strong band with methylated primers, while DNA from the HL60 cell line generated a strong band with unmethylated primers. None of the patients revealed a methylated profile for *CDKN2A* (Fig S4), suggesting that methylation was not an alternative mode of *CDKN2A* inactivation in these cases.

### NGS data

Due to the paucity of available DNA from most infants we were unable to perform NGS on all cases and consequently prioritized those cases for which Guthrie Cards were available.

Patient BR4 presented an ETP‐profile with no typical T‐ALL molecular alterations. We performed whole exome sequencing (WES) on DNA isolated from diagnostic material, however this patient did not achieve remission and therefore no germline material was available for matched analyses. In order to uncover somatic alterations acquired by the leukaemic clone, we filtered out mutations listed in the dbSNP and/or 1000genomes databases (http://www.ncbi.nlm.nih.gov/snp/; http://www.1000genomes.org/) and identified a total of 832 single nucleotide variations (SNVs) and 872 insertions or deletions (indels) at diagnosis.

**Figure 3:**
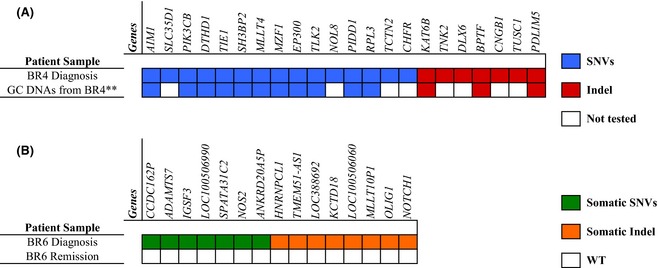
Principal mutations detected by next generation sequencing in patients BR4 and BR6. (A) List of the relevant genes affected by mutations (SNVs and indels) for BR4 at diagnosis. Guthrie card DNAs (GC) from patient BR4 were not submitted to WES analysis but were subsequently used to check the somatic *versus* germline status of the mutations detected at diagnosis. (B) Somatic alterations (SNVs and indels) detected by WGS in BR6 diagnostic DNA. A remission (germline) sample confirmed the somatic status of all the alterations listed. For both (A) and (B), we highlight the mutations affecting coding areas only. Different colours discriminate SNVs (WES‐blue, WGS‐green) from indels (WES‐red, WGS‐orange). **WES was not performed on this material. Guthrie card DNAs were investigated by conventional Sanger sequencing. SNV, single nucleotide variation; indel, insertion or deletion; WES, whole exome sequencing; WGS, whole genome sequencing; WT, wild‐type.

In the BR4 diagnostic sample, after respectively filtering the data by read depth (between 20–250x), coding areas only and deleterious/possibly damaging at protein level (VEP‐Ensembl; http://www.ensembl.org/Homo_sapiens/Tools/VEP), we detected 176 SNVs and 272 indels. We decided to focus particularly on 22 affected genes, which have an established causal role in oncogenesis and could therefore be considered as ‘drivers’ of leukaemia. For SNVs we chose *AIM1*,* SLC35D1*,* PIK3CB*,* DTHD1*,* TIE1*,* SH3BP2*,* MLLT4*,* MZF1*,* EP300*,* TLK2*,* NOL8*,* PIDD1*,* RPL3*,* TCTN2* and *CHFR* and for indels we chose; *KAT6B*,* TNK2, DLX6, BPTF*,* CNGB1*,* TUSC1* and *PDLIM5* (Fig 3A and Table SV). Due to the paucity of available patient material, we simply confirmed selected heterozygous point mutations and indels by Sanger sequencing in 14 out of the 22 chosen genes, i.e. *AIM1*,* PIK3CB*,* DTHD1*,* TIE1*,* SH3BP2*,* MLLT4*,* MZF1*,* EP300*,* TLK2*,* PIDD1*,* RPL3*,* KAT6B*,* BPTF* and *PDLIM5* (data not shown).

For patient BR6 we used a whole genome sequencing (WGS) approach to precisely determine the CNAs breakpoints with the aim of using this data for subsequent blood spot backtracking analyses. We filtered the WGS data by somatic alterations only, somatic *P *≤ 0·05 and exonic areas only and identified 145 mutations (SNVs and indels). Here, we particularly highlight 15 genes that were affected by novel mutations, i.e. mutations not been previously described in reference databases. The genes were: *CCDC162P*,* ADAMTS7*,* IGSF3*,* LOC100506990*,* SPATA31C2*,* NOS2*,* ANKRD20A5P*,* HNRNPCL1*,* TMEM51‐AS1*,* LOC388692*,* KCTD18*,* LOC100506060*,* MLLT10P1*,* OLIG1* and *NOTCH1* (Fig 3B and Table SVI). Table 2 summarises the main genomic abnormalities observed in our rare series of iT‐ALL.

**Table 2:** Main genomic findings observed in our series of infant T‐cell acute lymphoblastic leukaemia cases

Patient ID	Main CNAs	Gene Alterations
BR1	*CDKN2A/B* del	*NOTCH1* and *FBXW7* mutations
BR2	*IKZF1* del	*KMT2A‐MLLT1*
	*ETV6* del	
	*FLT3* del	
BR3	*11p13del/LMO2* over	
BR4	*MLF1* del	*AIM1, SLC35D1, PIK3CB, DTHD1, TIE1,*
		*SH3BP2, MLLT4, MZF1, EP300, TLK2,*
		*NOL8, PIDD1, RPL3, TCTN2, CHFR, KAT6B,*
		*TNK2, DLX6, BPTF, CNGB1, TUSC1* and *PDLIM5*
BR5	*11p13del/LMO2* over	
	*RB1* del	
BR6	*MLF1* del	*KMT2A‐MLLT1*
	*PTEN* del	*NOTCH1* mutation
BR7	*MLF1* del	
UK1	*MLLT4* del	*KMT2A‐MLLT4*
	*KMT2A* del	
FR1	*CDKN2A/B* del	*PTEN* mutation
FR2	*CDKN2A/B* del	
FR3	*CDKN2A/B* del	*NOTCH1* and *PTEN* mutations
FR4	*11q14‐q23del/ATM* and *EED*	*NOTCH1* mutation
FR5	*CDKN2A/B* del	*FBXW7* mutation

ID, identification; CNAs, copy number alterations; del, deletion; over, overexpression.

### Backtracking aberrations to an origin in utero

We also sought to investigate the early onset of genetic abnormalities by backtracking to birth the aberrations already present at diagnosis. We obtained archived Guthrie cards of four of the patients (BR4, BR5, BR6 and BR7). Potential clonal markers among these four patients included: a rearranged *TRD* (BR7); a *KMT2A‐r,* a *PTEN* deletion and a *NOTCH1* indel (all in BR6); and 11p13 and *RB1* deletions (both in BR5). The fourth case (BR4) harboured 22 mutations affecting cancer‐associated genes and a *MLF1* deletion as its only CNA.

**Figure 4:**
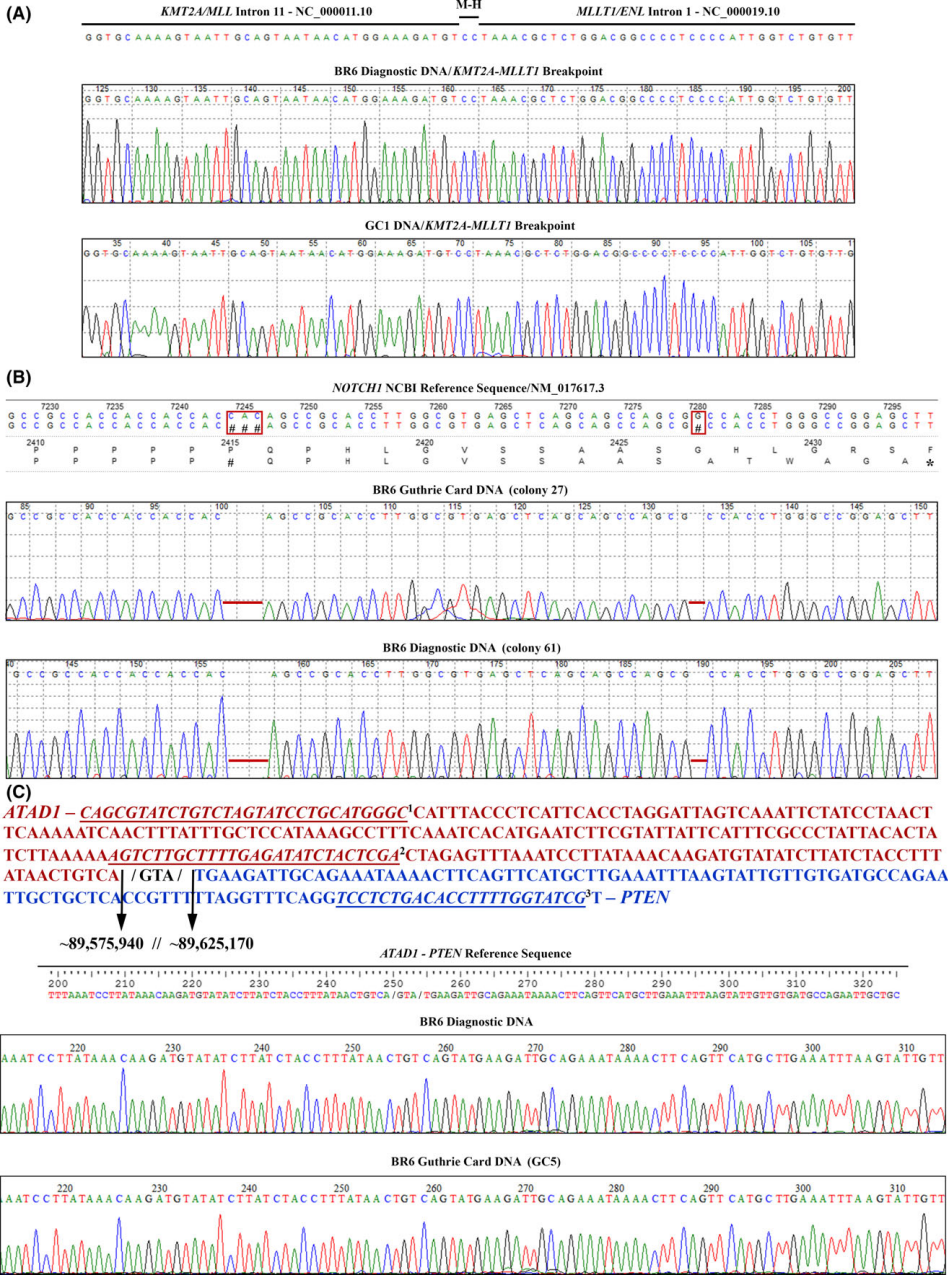
Prenatally acquired alterations in patient BR6. (A) *KMT2A‐MLLT1* Sanger sequencing of BR6 diagnostic and Guthrie card DNAs, showing that the patient presented an identical *KMT2A‐MLLT1* breakpoint at both time points. Top panel: GRCh38 Primary Assembly as reference for both gene sequences (*KMT2A*/NC_000011·10 and *MLLT1*/NC_000019·10). M‐H = micro‐homologies. (B) Cloning experiment detecting *NOTCH1*‐PEST deletion in the Guthrie card. The top sequence shows the *NOTCH1* NCBI Reference Sequence – NM_017617·3 – used to locate both mutations. The base pairs and amino acids deleted are highlighted in the red boxes. The first box represents sequenced Guthrie card DNA (colony 27) from patient BR6 harbouring both *NOTCH1*‐PEST deletions, i.e. c.7244_7246delCAC and c.7280delG and the second box shows BR6 diagnostic DNA (colony 61) also presenting both deletions. The c.7244_7246delCAC causes a Proline (P) deletion (# in the protein sequence p.2415delP), whereas c.7280delG generates a premature stop codon (*). **(C)** *PTEN* deletion breakpoint in patient BR6 diagnostic and Guthrie card DNAs. Primers 1 and 3 were used for the first PCR reaction and 2 and 3 for the semi‐nested one. Sanger sequencing data shows the deletion breakpoint present in both diagnostic (top, BR6 Diagnostic DNA) and also in one of ten Guthrie card DNAs tested (bottom, BR6 Guthrie card DNA‐GC5). GRCh37/hg19 was the reference for these analyses.

We previously determined *MLLT1* as the partner for *KMT2A‐r* in patient BR6 and subsequently the breakpoint sequence of this rearrangement (Emerenciano *et al*, 2013), which allowed us to design patient‐specific primers to interrogate this rearrangement in the Guthrie card DNAs. From ten individual Guthrie card DNAs examined for BR6, one was *KMT2A‐MLLT1* ^*+*^ (Fig 4A). To investigate the prenatal origin of the *NOTCH1* indel detected at diagnosis we also performed a specific semi‐nested PCR experiment. In total, we analysed 1200 cloned sequences from the ten blood spot DNAs and identified a single clone with the same *NOTCH1*‐PEST deletion that was present at diagnosis (c.7280delG). Unexpectedly, this clone also harboured a new mutation found 36 base pairs upstream from c.7280delG, (i.e. c.7244_7246delCAC, Fig 4B). This 3 base‐pair CAC deletion was also detected alone in 15/1200 Guthrie card clones. Although this deletion was not initially discovered in the bulk DNA analysis of diagnostic material, after cloning the *NOTCH1*‐PEST amplicon from BR6 diagnostic DNA, we found 1/100 clones with both c.7244_7246delCAC and c.7280delG (Fig 4B). Furthermore, as expected for a heterozygous mutation, we observed 50/100 clone sequences with the c.7280delG only. These results suggest that *NOTCH1* c.7280delG and c.7244_7246delCAC both occurred prenatally and that the latter potentially occurred (independently) in a cell that did not represent the major clone at diagnosis.

We used WGS to determine the precise breakpoint for the *PTEN* deletion detected by SNP‐array in the BR6 diagnostic sample. The breakpoint in this diagnostic material was cloned using specific primers designed from the WGS coordinates (Fig 4C). Subsequently, we used a semi‐nested approach to interrogate the ten DNAs from the Guthrie cards of this patient. One positive DNA (GC5) was identified and Sanger sequenced, confirming that the *PTEN* deletion in the diagnostic sample was present at birth (Fig 4C). To our knowledge this is the first observation of a gene deletion being identified in a neonatal blood spot.

We next tested the blood spot DNAs from patient BR4 by conventional PCR for the 14 WES mutations validated in the diagnostic DNA. Unfortunately, this patient did not achieve remission and died shortly after diagnosis. All 14 mutations were found in the four Guthrie card DNAs tested (Fig 4A), thus confirming their presence before birth. Nevertheless, we cannot confirm whether these were acquired as somatic mutations *in utero* solely in haematopoietic cells or were indeed germline mutations.

For patient BR5 we were unable to clone the deletion breakpoints for 11p13 (Fig S1) or for a large heterozygous deletion on chromosome 13 downstream of exon 1 in the tumour suppressor gene *RB1* (Fig S2A). However, using Q‐PCR copy number analysis (Figs S2B and C) we were able to detect the loss of intron 17 of *RB1* in diagnostic and BR5 Guthrie card DNA while intron 1 was undeleted in both. The heterozygous deletion was variably detected in all Guthrie DNAs tested, but not in remission DNA, again suggesting a potential *in utero* origin for this aberration.

Finally, the *TRD* rearrangement identified in patient BR7 contained a very small V(N)J junction which, although found to be present in its neonatal blood spot, could not be confirmed as being patient‐specific with adequate specificity.

## Discussion

A novel finding in this unique series of iT‐ALL was the loss of 3q25·32 resulting in the complete deletion of *MLF1*, not previously described in T‐ALL nor in acute leukaemias as a deletion. In addition, we have interrogated over 90 European and Brazilian cases of childhood and adolescent T‐ALL and we were not able to detect this deletion (unpublished data). These data support the notion that deletion of *MLF1* may represent a specific marker of iT‐ALL. This gene was originally identified as a partner of *NPM1* in the translocation t(3;5)(q25;q34), commonly found in acute myeloid leukaemia (AML) and myelodysplastic syndromes and has been shown to play a key role in the leukaemogenesis of these neoplasias (Bras *et al*, 2012). *MLF1* plays a regulatory role in TP53 activity, stabilizing the protein by suppressing its E3 ubiquitin ligase (*RFWD2*) (Yoneda‐Kato *et al*, 2005). Based on these findings we suggest that this gene deletion may have an oncogenic function because TP53 degradation is triggered by E3 ubiquitin ligase activity in the absence of *MLF1*. Hence, we hypothesize that *MLF1* could act as a recurrent tumour suppressor gene in iT‐ALL, however functional studies will be needed to elucidate its role in leukaemogenesis.

We provide evidence that iT‐ALL, in common with infant pro‐B ALL (Ford *et al*, 1993; Gale *et al*, 1997) and at least some cases of T‐ALL in childhood (Ford *et al*, 1997; Eguchi‐Ishimae *et al*, 2008), can be initiated *in utero*. Definitive data was obtained on one patient (BR6). The neonatal blood spots archived for BR6 harboured the clonotypic *KMT2A‐MLLT1* fusion sequence, as well as the *NOTCH1* mutation and the *PTEN* deletion, albeit at low frequencies. Additionally, we found evidence suggesting independent deletions in the PEST domain of *NOTCH1*. Given the very young age of the patient this is not surprising, nevertheless these data suggest not only a prenatal initiation of iT‐ALL but significant clonal evolution prior to birth, i.e. sequential acquisition of several mutations. This might help explain the presentation features of high white cell count but very young age (BR6, 7 months). In contrast, for children who present with *ETV6‐RUNX1* pre‐B ALL at an older age, usually with low or modest leukaemic burdens, the fusion gene appears to be the only prenatal ‘driver’ event (Ma *et al*, 2013).

Our series of iT‐ALL displayed a lower frequency of all the major recurrent gene alterations and CNAs found in childhood T‐ALL (Mullighan *et al*, 2007; Andersson *et al*, 2015). *PTEN* mutations and/or deletions appeared to be the only abnormality that occurred with the expected frequency (9‐20%) (Gutierrez *et al*, 2009; Mendes *et al*, 2014). We also observed a lower frequency of alterations affecting *CDKN2A* than has been found in childhood leukaemia, suggesting that its role in iT‐ALL leukaemogenesis may be less important than in childhood T‐ALL or B lineage ALL (Mullighan *et al*, 2007). An absence of CNAs was recently reported in *KMT2A‐r* infant ALL (Andersson *et al*, 2015).

Previous literature reported a single case with concomitant *KMT2A* deletion and *KMT2A‐MLLT4* rearrangement, these abnormalities both being present in the diagnostic sample of a three‐year‐old child with T‐ALL (De Braekeleer *et al*, 2010). Therefore, patient UK1 is the second reported T‐ALL case with these *KMT2A* alterations combined. Deletion of *KMT2A* has also been described in cases of precursor B infant ALL with *KMT2A‐r* (Andersson *et al*, 2015).

A deletion in the classical tumour suppressor gene, *RB1*, was observed in one (BR5) of our 13 cases. *RB1* deletions are described at a frequency of 6‐10% for both B‐cell precursor ALL and T‐ALL in children and adults (Okamoto *et al*, 2010; Schwab *et al*, 2013). Deletion at 11p12p13 can act to transcriptionally activate the *LMO2* gene, a classic oncogene in T‐cell leukaemogenesis (Lecuyer & Hoang, 2004). Deletions affecting locus 11p13 were identified in two of our cases, including BR5, suggesting that this deletion could lead to *LMO2* activation and contribute to the development of iT‐ALL. Unfortunately, cells were not available from these patients to investigate *LMO2* expression. The 11q14·1‐q23·2del Chr11q/*ATM* deletions present in patient FR4 have been reported in 30% of chronic lymphocytic leukaemia cases (Edelmann *et al*, 2012; Skowronska *et al*, 2012) and at a lower rate in ALL (Schwab *et al*, 2013).

Patient BR4 presented an ETP‐profile with no typical T‐ALL molecular alterations. Hence, we performed WES analyses with a view to uncovering potential ‘driver’ alterations that could account for the emergence of leukaemia. By grouping our 22 highlighted genes according to the hallmarks of cancer (Hanahan & Weinberg, 2011), we uncovered roles including sustaining proliferative signalling, activating invasion and metastasis, resisting cell death and evading growth suppressors. Furthermore, three groups of mutations characteristic of ETP‐ALL (RAS signalling, haematopoietic and epigenetic regulators) (Zhang *et al*, 2012) were also observed in our case. Some of the aberrant genes that we uncovered do not yet have their functions fully elucidated. Given the ease of detection of mutations in the neonatal blood spots of this patient, compared to our other cases, we speculate that they may be germline mutations. It is of interest that an excess of germline variations in *KMT2A‐r* negative infant leukaemia has been described by Valentine *et al* (2014), who suggested that such cases may well be enriched for rare coding and deleterious germline variations in cancer‐associated genes. The authors postulate that such variations might comprise some proportion of the expected functional imbalance characteristically observed in cancer. This notion aligns with our own *KMT2A‐r* negative case that also did not reveal any of the expected ETP somatic mutations, such as *SETD2* or *EZH2*. Nevertheless *EP300,* a known ‘driver’ for ETP‐ALL (Zhang *et al*, 2012), was carried as a suspected germline mutation in patient BR4.

In summary, we have analysed the genomic abnormalities in a unique series of a rare subtype of paediatric leukaemia – T‐ALL in infants. The genotypes or mutational spectra are varied but, overall, different from those of T‐ALL in older children and adults. A novel aberration (for acute leukaemia), *MLF1* deletion, was present as a recurrent abnormality in three of 13 cases. Finally, we have provided evidence that some of the genetic abnormalities, including a *PTEN* deletion, were accrued prenatally.

## Author contributions

MBM: designed the study, conducted and analysed all the experiments and wrote the paper; FWvD: supervised all SNP procedures and analyses; SMC: assisted in sample preparation and supervised the FISH experiments; CLF and ME: performed FISH and/or molecular investigations; HK, EC, HC, JS: provided clinical samples and immunophenotypic/cytogenetic/clinical data; JG: performed the WGS analyses; MSPO and MG: designed the study and wrote the paper; AMF: designed and supervised the study, generated and analysed experimental data and wrote the paper. All authors critically reviewed and approved the final draft of the manuscript.

## Conflict of interest

The authors declare no conflict of interest.

## Supporting information


**Data S1.** Materials and methods.
**Data S2.** Supplementary tables with additional data.

**Fig S1.** Copy number analysis of chromosome 11 in our iT‐ALL series.

**Fig S2.** Genomic copy number analysis of *RB1*.

**Fig S3.** *CDKN2A* Q‐PCR copy number data of the Brazilian patients.

**Fig S4.** Methylation specific PCR (MSP) in our iT‐ALL cohort and two control cell lines.

**Table SI.** Clinical–demographic data of infant T‐ALL cases.

**Table SII.** Mutation screening data of infant T‐ALL cases.

**Table SIII.** SNP‐array copy number data of infant T‐ALL cases.

**Table SIV.** Contrast quality control (QC) data from SNP6·0‐arrays of the infant T‐ALL cases.

**Table SV.** WES data details for patient BR4.

**Table SVI.** WGS data details for patient BR6.
