# Supplementary material for: Distinctive genotypes in infants with T‐cell acute lymphoblastic leukaemia
Source: Br J Haematol. 2015 Jul 24;171(4):574–84. doi: 10.1111/bjh.13613 (PMC4737125; doi:10.1111/bjh.13613)
Supplement: Supplementary file 1 — Data S1. Materials and methods. Data S2. Supplementary tables with additional data. [file BJH-171-574-s001.doc]

**Supporting information: “Distinctive genotypes in infants with T-cell acute lymphoblastic leukaemia”**

**Data S1. Materials and methods**

*Patients*

Diagnostic samples were obtained from the Paediatric Haematology-Oncology Program, Instituto Nacional de Câncer (INCA), Rio de Janeiro - Brazil; Great Ormond Street Hospital, London - United Kingdom; Robert Debré Hospital, Paris - France and Saint-Louis Louis Hospital, Paris – France and screened for biomarkers diagnostic of iT-ALL . Informed consent was obtained from all subjects in accordance with the Declaration of Helsinki and ethics was approved by each participating centre. The research ethics committee from INCA approved the iT-ALL sample and data collection (CEP-INCA #107/06, #117/12 & CONEP #888.277). A summary of clinical-demographic data of our series of iT-ALL is shown in Table SI.

Table SI. Clinical-demographic data of infant T-ALL cases

| ***Patient ID*** | ***Age*** | ***Gender*** | ***WBC x10^9^/L (x109/L)*** | ***Blast %*** | ***Mediastinal Mass*** | ***SCT*** | ***Treatment Protocol**** | ***Outcome*** |
| --- | --- | --- | --- | --- | --- | --- | --- | --- |
| ***BR1*** | 12m | Male | 80.0 | 80 | No | Yes | GBTLI-1999 | Deceased |
| ***BR2*** | 8m | Male | 74.1 | 60 | No | Yes | GBTLI-1993 | Deceased |
| ***BR3*** | 6m | Female | 131.6 | 89 | Yes | NA | BFM-1995 | Deceased |
| ***BR4*** | 7m | Female | 53.2 | 78 | No | No | BFM-2002 | Deceased |
| ***BR5*** | 11m | Male | 65.8 | 80 | No | No | INTERFANT-2006 | Deceased |
| ***BR6*** | 7m | Female | 362.6 | 94 | No | Yes | GBTLI-1999 | Alive in CCR |
| ***BR7*** | 8m | Male | 21.6 | 43 | No | No | BFM-2002 | Deceased |
| ***UK1*** | 9m | Male | 236.1 | 80 | No | NA | INTERFANT-2006 | Deceased |
| ***FR1*** | 9m | Female | 165.0 | 82 | Yes | Yes | INTERFANT-2006 | Alive in CCR |
| ***FR2*** | 11m | Female | 150.0 | 95 | No | No | INTERFANT-1999 | Deceased |
| ***FR3*** | 12m | Male | 380.0 | 100 | No | No | INTERFANT-2006 | Deceased |
| ***FR4*** | 11m | Female | 60.0 | 74 | No | Yes | EORTC-58951 | Deceased |
| ***FR5*** | 9m | Female | 290.0 | 95 | No | Yes | EORTC-58081 | Alive in CCR |

Abbreviations: ID = identification; m = months; WBC = white blood cells count; CCR = complete continuous remission; SCT = stem cell transplant; NA = data not available. *Brazilian T-ALL patients were treated outside of clinical trials. Thus, all patients were treated according to previously published protocols with risk-adapted strategies for infant ALL .

*Leukaemia characterization*

In all cases, diagnosis of leukaemia was established by the morphology of lymphoid cells and immunophenotyping by flow cytometry using a previously established panel of monoclonal antibodies . The immunological classification of T-ALL was performed according to the EGIL criteria .

*DNA purification*

Genomic DNA from all iT-ALL cases was extracted from BM aspirate and/or PB samples using either: the QIAamp® DNA Blood Mini Kit (QIAGEN) or ethanol precipitation from the aqueous phase after phenol extraction. Guthrie card DNAs were extracted using the QIAamp DNA Micro Kit following the manufacturer's instructions (QIAGEN).

*Genome mapping analysis, copy number and LOH analysis*

Mapping analysis was performed using 500ng of DNA from matched samples (diagnosis/remission) for each patient. DNA was prepared according to the manufacturer's instructions using the Genome-Wide SNP Array 6.0 assay protocol (Affymetrix). SNP genotypes were obtained using Affymetrix GCOS v1.4 software and Affymetrix GTYPE v4.0 software with the BRLMM algorithm. Matched samples were analyzed with Partek Genomics Suite 6.6 (Partek®) and CNAG 3.3.0.0-beta (http://plaza.umin.ac.jp/genome/) to determine copy number and LOH . Positions of regions of LOH and gain were identified via the University of California Santa Cruz Genome Browser (Hg18, March 2006 Assembly; <http://genome.ucsc.edu/cgi-bin/hgGateway>). SNP6.0 array files are available at GEO database #GSE67271.

*FISH*

Interphase FISH was performed on archival viable cells from available cases using standard methods. FISH for the *KMT2A-r* was performed with a commercial LSI *MLL* Dual Colour, Break Apart Rearrangement probe (Vysis), according to manufacturer’s instructions. Bacterial Artificial Chromosome (BAC) or fosmid probes for other regions of interest (*CDKN2A*, *MLF1*, *PTEN*, *KMT2A*, *MLLT4;* Supplemental Methods) were obtained from the BACPAC Resource Centre (http://bacpac.chori.org). Labelling of in-house probes, hybridization, washes and analysis were all performed according to standard protocols . In each case, at least 100 nuclei were scored for each probe, cut-off levels for each probe were established using normal control metaphase spreads.

The following FISH clones were obtained from the BACPAC Resource Centre (http://bacpac.chori.org) for regions of interest**:** *CDKN2A/B*, *MLF1*, *PTEN*, *KMT2A* (also known as *MLL*) and *MLLT4* (also known as *AF6*)*.*

| **Our probe ID** | **BACPAC Resource clone ID** | **Target Gene** |
| --- | --- | --- |
| *CDKN2A.1* | G248P82010F5 | *CDKN2A* |
| *CDKN2A/B.2* | G248P82557D2 | *CDKN2A/B* |
| *MLF1.1* | G248P82424F3 | *MLF1* |
| *MLF1.2* | G248P80353A3 | *MLF1* |
| *PTEN2* | G248P80986D1 | *PTEN* |
| *PTEN3* | G248P85191D2 | *PTEN* |
| *MLL.b* | G248P84267F9 | *KMT2A* |
| *AF6* | RP3-470B24 | *MLLT4/AF6* |

*Copy number assays used in the quantitative real-time PCR experiments*

To confirm the copy number alterations (CNA) initially defined by Genome-Wide SNP Array 6.0, we performed DNA quantitative real-time PCR (Q-PCR) using commercial TaqMan® DNA copy number assays (Applied Biosystems®) to target *CDKN2A* and *RB1*; human RNaseP was used as a reference. *CDKN2A* was investigated in all thirteen iT-ALL diagnostic DNAs and *RB1* specifically on BR5 diagnostic and Guthrie card DNAs. Data analyses were performed with CopyCaller® Software (Applied Biosystems®).

| **Our probe ID** | **Applied Biosystems ID** | **Target Gene** |
| --- | --- | --- |
| *CDKN2A*-1 | Hs03724208_cn | *CDKN2A* |
| *CDKN2A*-4 | Hs03704181_cn | *CDKN2A* |
| *CDKN2A*-5 | Hs03721302_cn | *CDKN2A* |
| *RB1*-Intron 1 | Hs07026395_cn | *RB1* |
| *RB1*-Intron 17 | Hs07034351_cn | *RB1* |
| *RNaseP*-reference | Catalog#4403326 | *RNaseP* |

*Next generation sequence – NGS*

Due to paucity of available DNA from most infants we were unable to perform NGS on all cases and consequently prioritized those cases for which Guthrie Cards were available.

Whole exome sequencing (WES) - Exome capture was performed on one case (BR4) using the Agilent SureSelect Human All Exon V5 kit following manufacturer’s procedures (Agilent) and sequenced by Illumina paired end sequencing (protocol v1.2). Samples were sequenced on a HiSeq 2000 (Illumina) 100 cycle run according to manufacturer’s protocols. Analysis was completed in Oxford Gene Technology’s exome pipeline; briefly, reads were aligned to the hg19/GRCh37 using Burrows-Wheeler Aligner (BWA) 0.6.2 . Local realignment was performed around indels (insertions and/or deletions) with the Genome Analysis Toolkit (GATK v1.6) IndelRealigner . Optical and PCR duplicates were marked in BAM files using Picard 1.107 (http://picard.sourceforge.net). Original HiSeq base quality scores were recalibrated using GATK Table Recalibration and per-sample variants called with GATK Unified Genotyper. Indels and SNVs were hard-filtered according to Broad Institute best-practice guidelines to eliminate false positive calls. Variant annotation was performed with a modified version of Ensembl Variant Effect Predictor (VEP) .

Whole genome sequencing (WGS) - WGS capture, to determine chromosome breakpoints, was performed on diagnostic and remission samples from patient BR6 using Illumina technology (paired-end whole genome sequencing). Samples were sequenced on a HiSeq2500 (Illumina) paired-end rapid run acquiring 2x100bp reads. Casava software (v1.8, Illumina) was used to make base calls and demultiplex the sequencing data. Sequences were output in fastq format and reads failing the Illumina chastity filter were removed before further analysis.

WGS data was aligned against hg19/GRCh37 using the BWA 0.6.2 aligner. Duplicate reads were removed using Picard. Depth of coverage of the genome was calculated with the BedTools package (v2.17.0) . Germline (remission) and leukemic (diagnostic) paired data were analysed to identify SNVs and small indels using Varscan 2.3.6 . Variants were filtered using the ‘somaticFilter’ command to remove clusters of false positives and SNV calls near indels and further processed using the ‘processSomatic’ command to provide ‘high confidence’ variants. Variants were then annotated using the Annovar software tool (v2013Aug23) . CNAs were called by the varscan ‘copynumber’ and ‘copyCaller’ commands using a data ratio of 1.915. Output was further processed using the DNAcopy R package implementing a Circular Binary Segmentation algorithm and plotting results for visualization. Regions harbouring CNAs previously identified by SNP6.0 and FISH analyses, but which Varscan had not identified, were interrogated by visualization of read pair arcs in the Savant browser (v2.0.4).

*KMT2A-MLLT1 gene fusion backtracking*

To backtrack the *KMT2A-MLLT1* fusion identified at diagnosis in case BR6, we used the previously determined rearrangement breakpoint to design patient specific PCR primers (available on request). With a nested PCR approach we tested ten individual DNAs extracted from different regions of BR6 neonatal blood spots. After PCR amplification the products were sequenced to confirm the presence of the *KMT2A-r* specific sequence.

*Promoter methylation status of CDKN2A*

Methylation specific PCR (MSP) of the *CDKN2A* promoter region was performed as described previously . Genomic DNAs were subjected to sodium bisulphite modification using the EpiTect Bisulfite Kit according to manufacturer’s instructions (QIAGEN). MSP was performed using unmethylated (U) and methylated (M) primers, following conditions previously described . All diagnostic samples were tested regardless of the *CDKN2A* SNP-array status. PCR products were run through 1.5% agarose stained with ethidium bromide, purified and analysed by Sanger sequencing.

**Data S2. Supplementary tables with additional data**

Table SII. Mutation screening data of infant T-ALL cases

Table SIII. SNP-array copy number data of infant T-ALL cases

Table SIV. Contrast quality control (QC) data from SNP6.0-arrays of the infant T-ALL cases

Table SV. WES data details for patient BR4

Table SVI. WGS data details for patient BR6

**Supporting Figures**

**
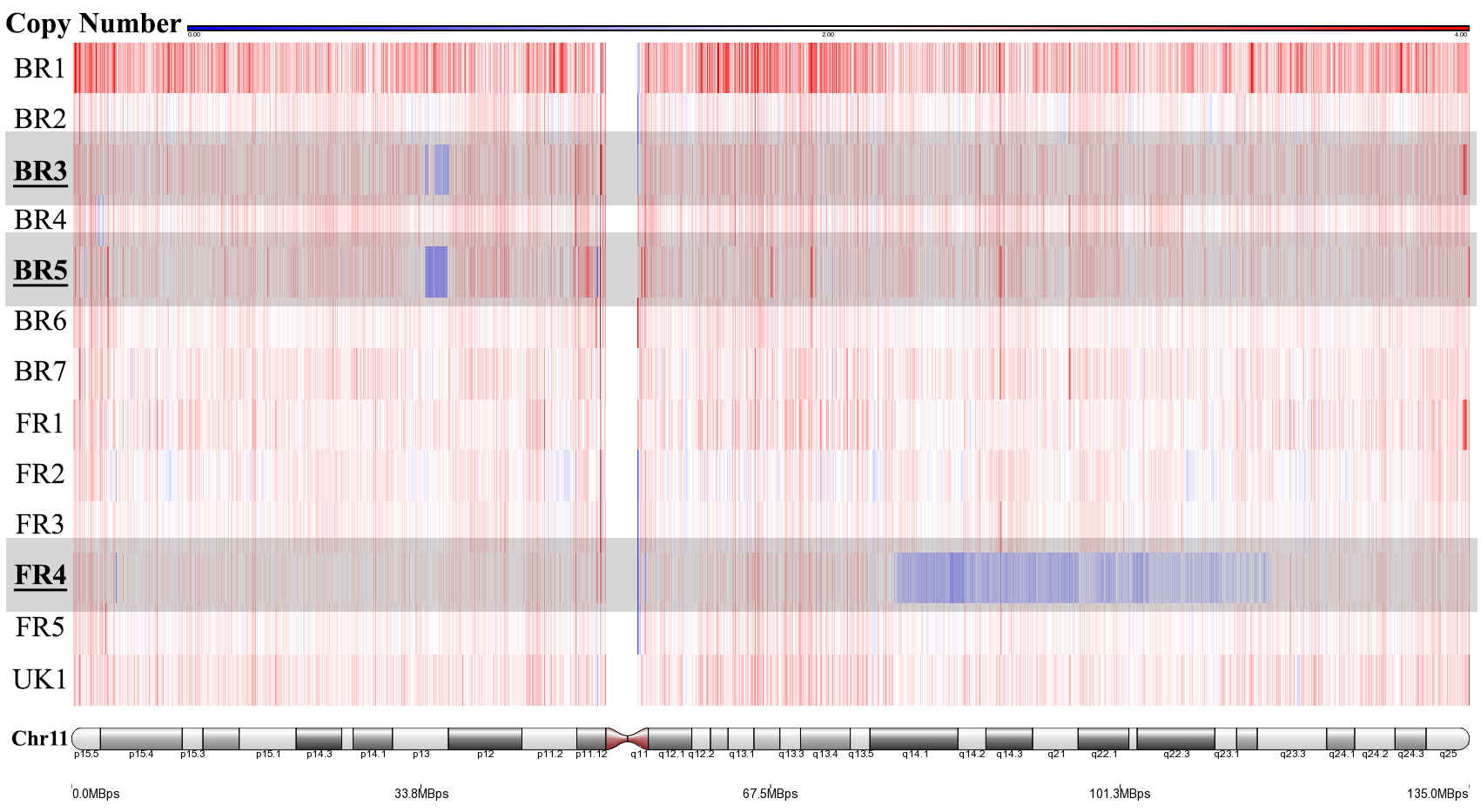
**

**Fig S1. Copy number analysis of chromosome 11 in our iT-ALL series.** The blue areas represent the deleted regions in the three infant cases (highlighted in the grey box), BR3 and BR5 deletions are *LMO2*-related (11p13) and FR4 CNA is *ATM*-related (11q14.1-q23.2).


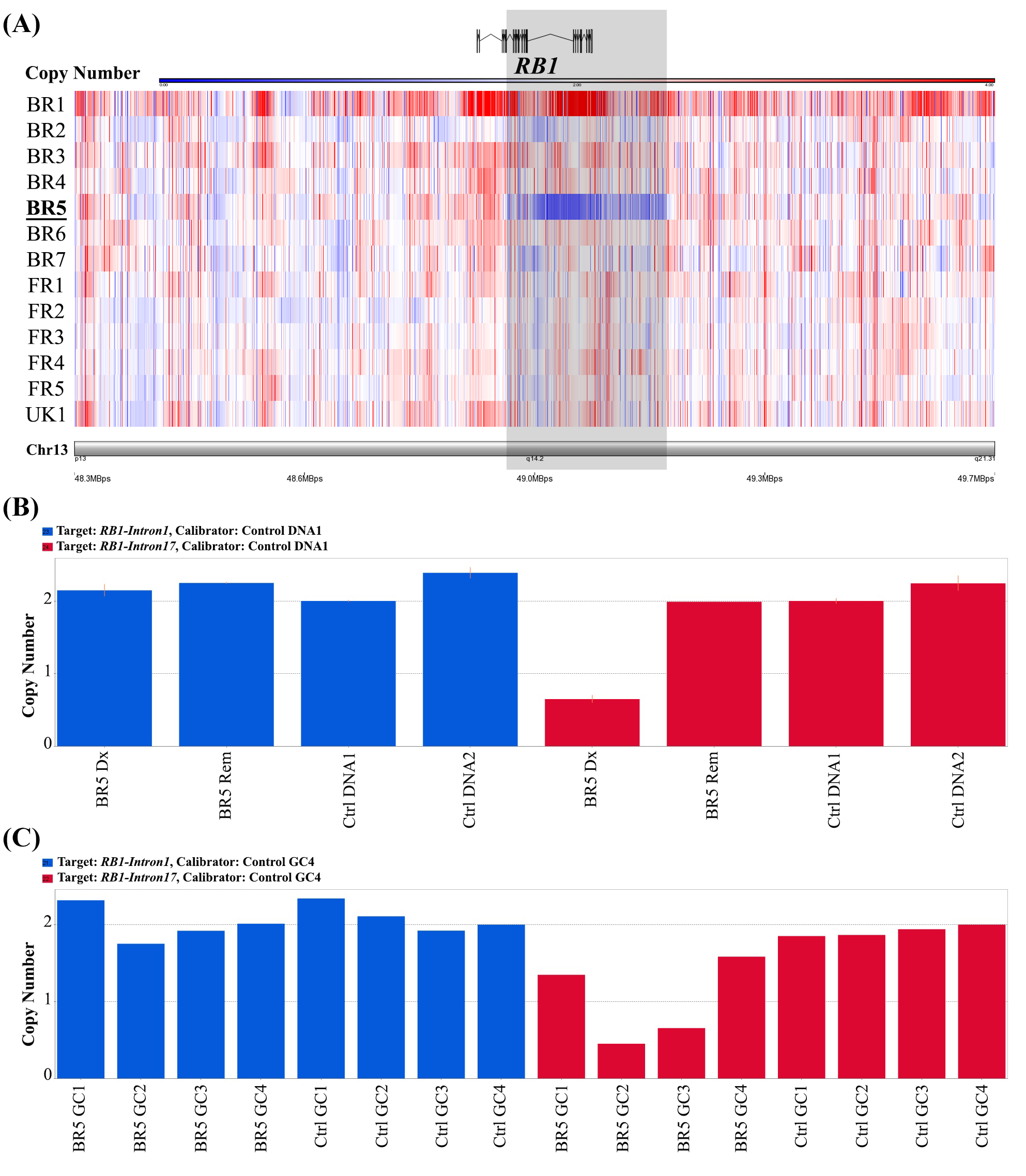


**Fig S2. Genomic copy number analysis of *RB1*.** **(A)** SNP-array heatmap of chromosome 13 including all iT-ALL. The *RB1* locus is highlighted and a deletion of *RB1* (blue) is seen for patient BR5. **(B) and (C)** Q-PCR copy number analysis with two different assays located in introns 1 and 17 of *RB1*. **(B)** The red bars show deletion of *RB1* (intron 17) for BR5 diagnostic DNA (BR5 Dx), while intron 1 (blue) is non-deleted (two copies). The matched remission sample (BR5 Rem) shows two copies of *RB1* for both probes. Two control DNAs (two *RB1* copies) were also included. **(C)** Q-PCR copy number data for Guthrie card DNAs showing *RB1* deletion in. Four control Guthrie card DNAs (all *RB1* WT) were also included*.*

**
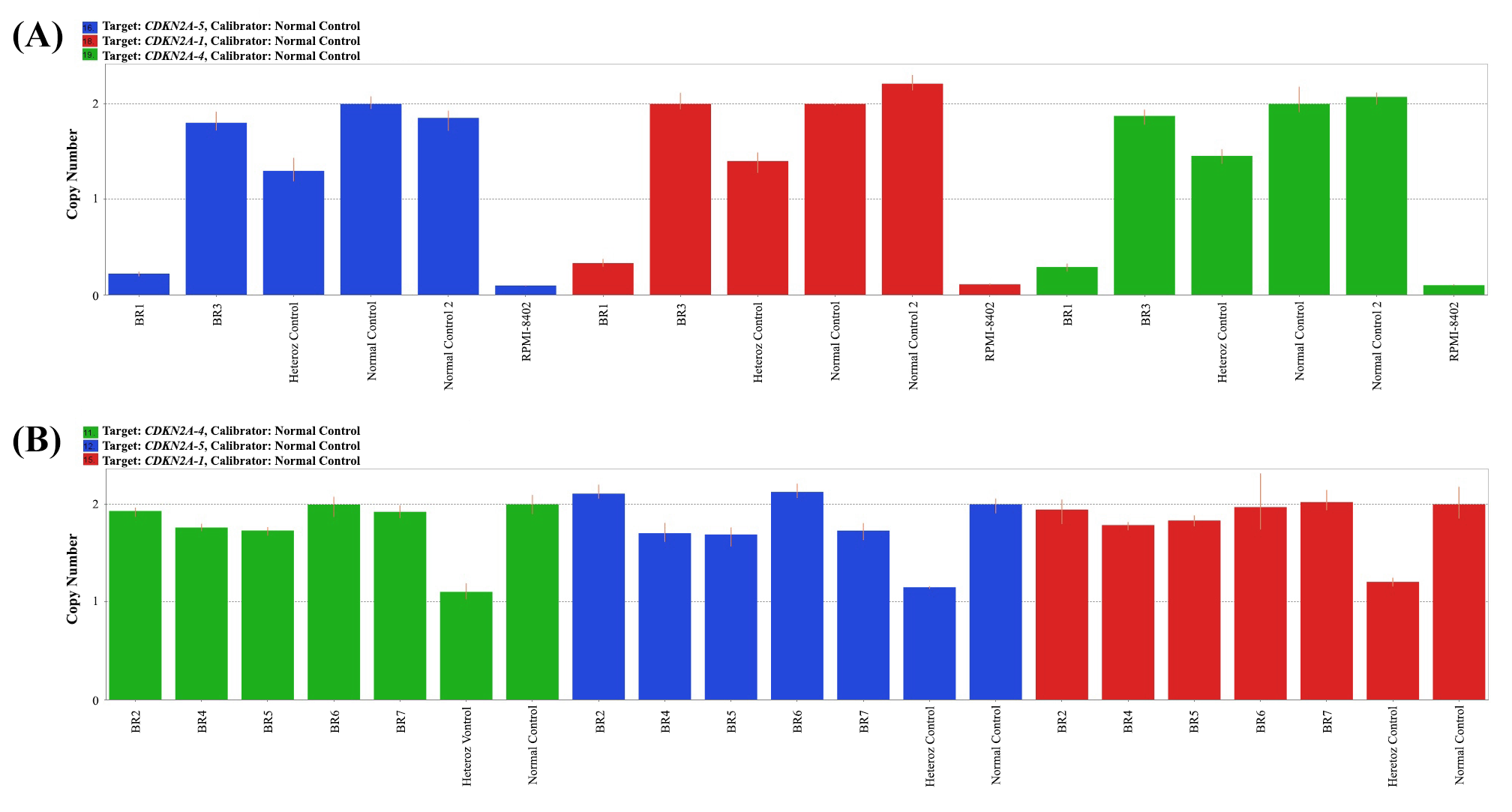
**

**Fig S3. *CDKN2A* Q-PCR copy number data of the Brazilian patients.** The graphs show Q-PCR copy number analysis with three different assays for *CDKN2A*. **(A)** The Q-PCR results confirmed the SNP data with zero *CDKN2A* copies for case BR1 and two *CDKN2A* copies for BR3. Also included in this experiment a heterozygous control, two normal controls and RPMI-8402 (*CDKN2A* homozygous deleted). **(B)** Q-PCR data for the other Brazilian cases, confirming the WT status of *CDKN2A* for BR2, BR4, BR5, BR6 and BR7. A heterozygous deleted and a WT controls were used in these analyses.

**
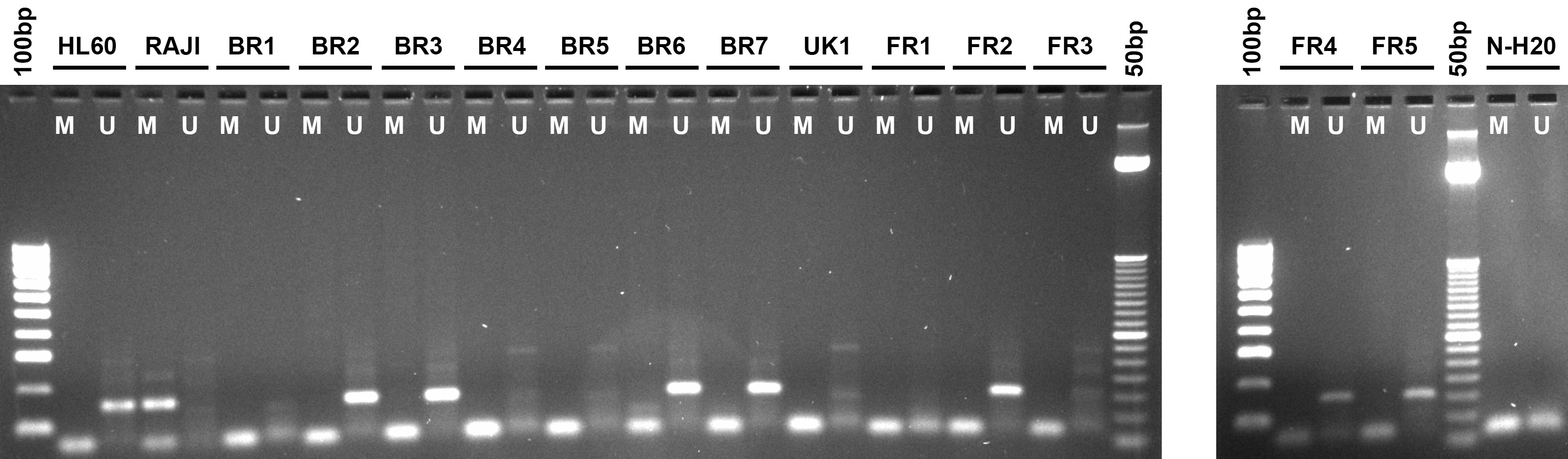
**

**Fig S4. Methylation Specific PCR (MSP) in our iT-ALL cohort and two control cell lines.** DNA from the HL60 cell line produced a strong band with unmethylated primers, while DNA from the RAJI cell line produced a strong band with methylated primers. None of the infant DNAs produced amplicons with methylated primers. DNAs from BR1 and FR3 did not produce amplicons, since those two cases had homozygously deleted *CDKN2A*.

**Supporting References**

Bateman, C.M., Colman, S.M., Chaplin, T., Young, B.D., Eden, T.O., Bhakta, M., Gratias, E.J., van Wering, E.R., Cazzaniga, G., Harrison, C.J., Hain, R., Ancliff, P., Ford, A.M., Kearney, L. & Greaves, M. (2010) Acquisition of genome-wide copy number alterations in monozygotic twins with acute lymphoblastic leukemia. *Blood,* **115,** 3553-3558.

Bene, M.C., Castoldi, G., Knapp, W., Ludwig, W.D., Matutes, E., Orfao, A. & van't Veer, M.B. (1995) Proposals for the immunological classification of acute leukemias. European Group for the Immunological Characterization of Leukemias (EGIL). *Leukemia,* **9,** 1783-1786.

Brandalise, S.R., Pinheiro, V.R., Aguiar, S.S., Matsuda, E.I., Otubo, R., Yunes, J.A., Pereira, W.V., Carvalho, E.G., Cristofani, L.M., Souza, M.S., Lee, M.L., Dobbin, J.A., Pombo-de-Oliveira, M.S., Lopes, L.F., Melnikoff, K.N., Brunetto, A.L., Tone, L.G., Scrideli, C.A., Morais, V.L. & Viana, M.B. (2010) Benefits of the intermittent use of 6-mercaptopurine and methotrexate in maintenance treatment for low-risk acute lymphoblastic leukemia in children: randomized trial from the Brazilian Childhood Cooperative Group--protocol ALL-99. *J Clin Oncol,* **28,** 1911-1918.

Emerenciano, M., Agudelo Arias, D.P., Coser, V.M., de Brito, G.D., Macedo Silva, M.L. & Pombo-de-Oliveira, M.S. (2006) Molecular cytogenetic findings of acute leukemia included in the Brazilian Collaborative Study Group of Infant acute leukemia. *Pediatr Blood Cancer,* **47,** 549-554.

Emerenciano, M., Meyer, C., Mansur, M.B., Marschalek, R., Pombo-de-Oliveira, M.S. & Brazilian Collaborative Study Group of Infant Acute, L. (2013) The distribution of MLL breakpoints correlates with outcome in infant acute leukaemia. *Br J Haematol,* **161,** 224-236.

Herman, J.G., Graff, J.R., Myohanen, S., Nelkin, B.D. & Baylin, S.B. (1996) Methylation-specific PCR: a novel PCR assay for methylation status of CpG islands. *Proc Natl Acad Sci U S A,* **93,** 9821-9826.

Kearney, L. & Colman, S. (2009) Specialized fluorescence in situ hybridization (FISH) techniques for leukaemia research. *Methods Mol Biol,* **538,** 57-70.

Koboldt, D.C., Zhang, Q., Larson, D.E., Shen, D., McLellan, M.D., Lin, L., Miller, C.A., Mardis, E.R., Ding, L. & Wilson, R.K. (2012) VarScan 2: somatic mutation and copy number alteration discovery in cancer by exome sequencing. *Genome Res,* **22,** 568-576.

Li, H. & Durbin, R. (2009) Fast and accurate short read alignment with Burrows-Wheeler transform. *Bioinformatics,* **25,** 1754-1760.

Mansur, M.B., Emerenciano, M., Brewer, L., Sant'Ana, M., Mendonca, N., Thuler, L.C., Koifman, S. & Pombo-de-Oliveira, M.S. (2009) SIL-TAL1 fusion gene negative impact in T-cell acute lymphoblastic leukemia outcome. *Leuk Lymphoma,* **50,** 1318-1325.

Mansur, M.B., Emerenciano, M., Splendore, A., Brewer, L., Hassan, R. & Pombo-de-Oliveira, M.S. (2010) T-cell lymphoblastic leukemia in early childhood presents NOTCH1 mutations and MLL rearrangements. *Leuk Res,* **34,** 483-486.

McKenna, A., Hanna, M., Banks, E., Sivachenko, A., Cibulskis, K., Kernytsky, A., Garimella, K., Altshuler, D., Gabriel, S., Daly, M. & DePristo, M.A. (2010) The Genome Analysis Toolkit: a MapReduce framework for analyzing next-generation DNA sequencing data. *Genome Res,* **20,** 1297-1303.

McLaren, W., Pritchard, B., Rios, D., Chen, Y., Flicek, P. & Cunningham, F. (2010) Deriving the consequences of genomic variants with the Ensembl API and SNP Effect Predictor. *Bioinformatics,* **26,** 2069-2070.

Nannya, Y., Sanada, M., Nakazaki, K., Hosoya, N., Wang, L., Hangaishi, A., Kurokawa, M., Chiba, S., Bailey, D.K., Kennedy, G.C. & Ogawa, S. (2005) A robust algorithm for copy number detection using high-density oligonucleotide single nucleotide polymorphism genotyping arrays. *Cancer Res,* **65,** 6071-6079.

Pieters, R., Schrappe, M., De Lorenzo, P., Hann, I., De Rossi, G., Felice, M., Hovi, L., LeBlanc, T., Szczepanski, T., Ferster, A., Janka, G., Rubnitz, J., Silverman, L., Stary, J., Campbell, M., Li, C.K., Mann, G., Suppiah, R., Biondi, A., Vora, A. & Valsecchi, M.G. (2007) A treatment protocol for infants younger than 1 year with acute lymphoblastic leukaemia (Interfant-99): an observational study and a multicentre randomised trial. *Lancet,* **370,** 240-250.

Quinlan, A.R. & Hall, I.M. (2010) BEDTools: a flexible suite of utilities for comparing genomic features. *Bioinformatics,* **26,** 841-842.

Stary, J., Zimmermann, M., Campbell, M., Castillo, L., Dibar, E., Donska, S., Gonzalez, A., Izraeli, S., Janic, D., Jazbec, J., Konja, J., Kaiserova, E., Kowalczyk, J., Kovacs, G., Li, C.K., Magyarosy, E., Popa, A., Stark, B., Jabali, Y., Trka, J., Hrusak, O., Riehm, H., Masera, G. & Schrappe, M. (2014) Intensive chemotherapy for childhood acute lymphoblastic leukemia: results of the randomized intercontinental trial ALL IC-BFM 2002. *J Clin Oncol,* **32,** 174-184.

Sulong, S., Moorman, A.V., Irving, J.A., Strefford, J.C., Konn, Z.J., Case, M.C., Minto, L., Barber, K.E., Parker, H., Wright, S.L., Stewart, A.R., Bailey, S., Bown, N.P., Hall, A.G. & Harrison, C.J. (2009) A comprehensive analysis of the CDKN2A gene in childhood acute lymphoblastic leukemia reveals genomic deletion, copy number neutral loss of heterozygosity, and association with specific cytogenetic subgroups. *Blood,* **113,** 100-107.

Wang, K., Li, M. & Hakonarson, H. (2010) ANNOVAR: functional annotation of genetic variants from high-throughput sequencing data. *Nucleic Acids Res,* **38,** e164.
