# Supplementary figures and images for: Distinctive genotypes in infants with T‐cell acute lymphoblastic leukaemia
Source: Br J Haematol. 2015 Jul 24;171(4):574–84. doi: 10.1111/bjh.13613 (PMC4737125; doi:10.1111/bjh.13613)

## Slide 1
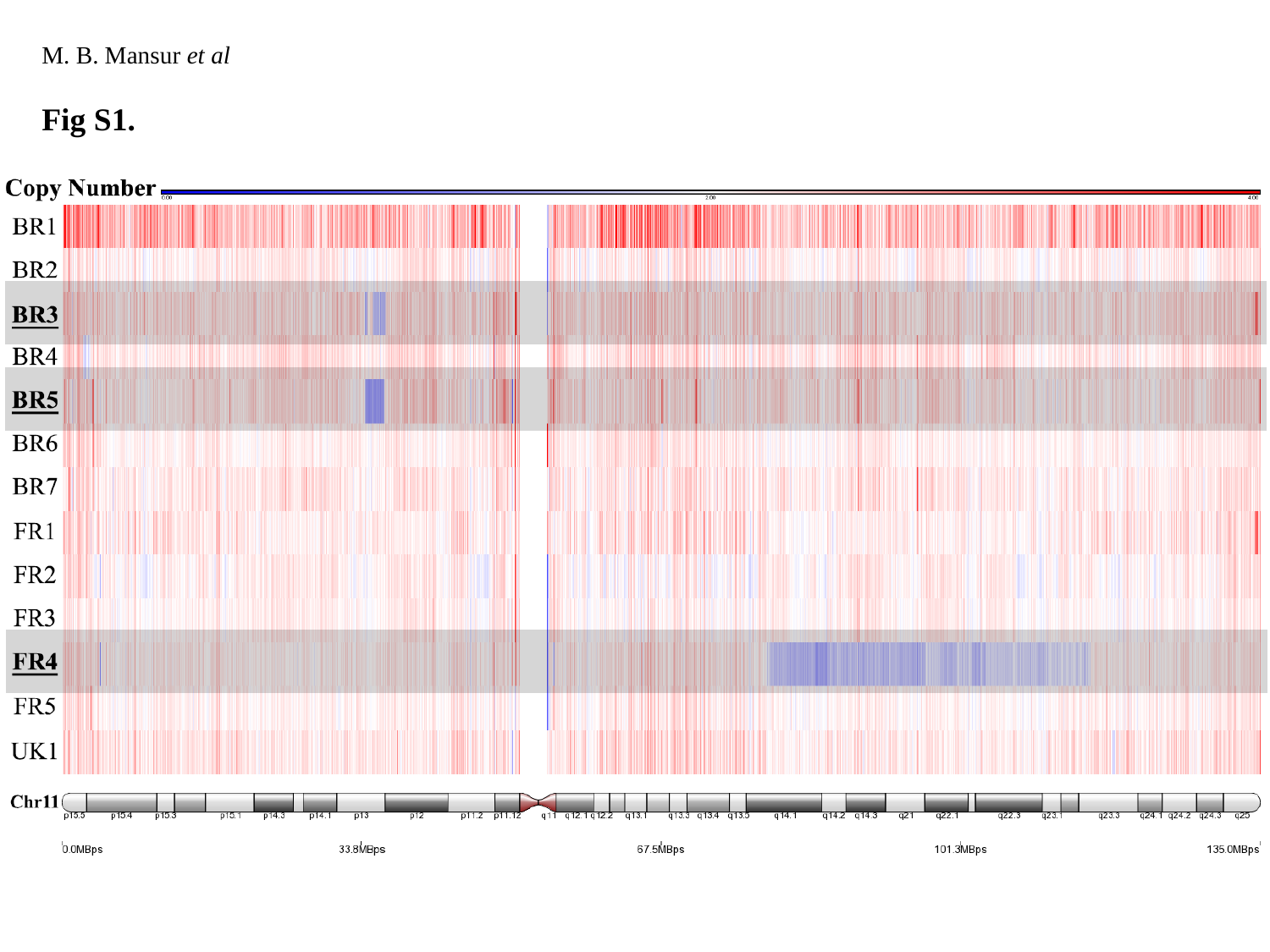

M. B. Mansur et al
Fig S1.

Supplement: Supplementary file 2 — Fig S1. Copy number analysis of chromosome 11 in our iT‐ALL series. [file BJH-171-574-s002.ppt]

## Slide 1
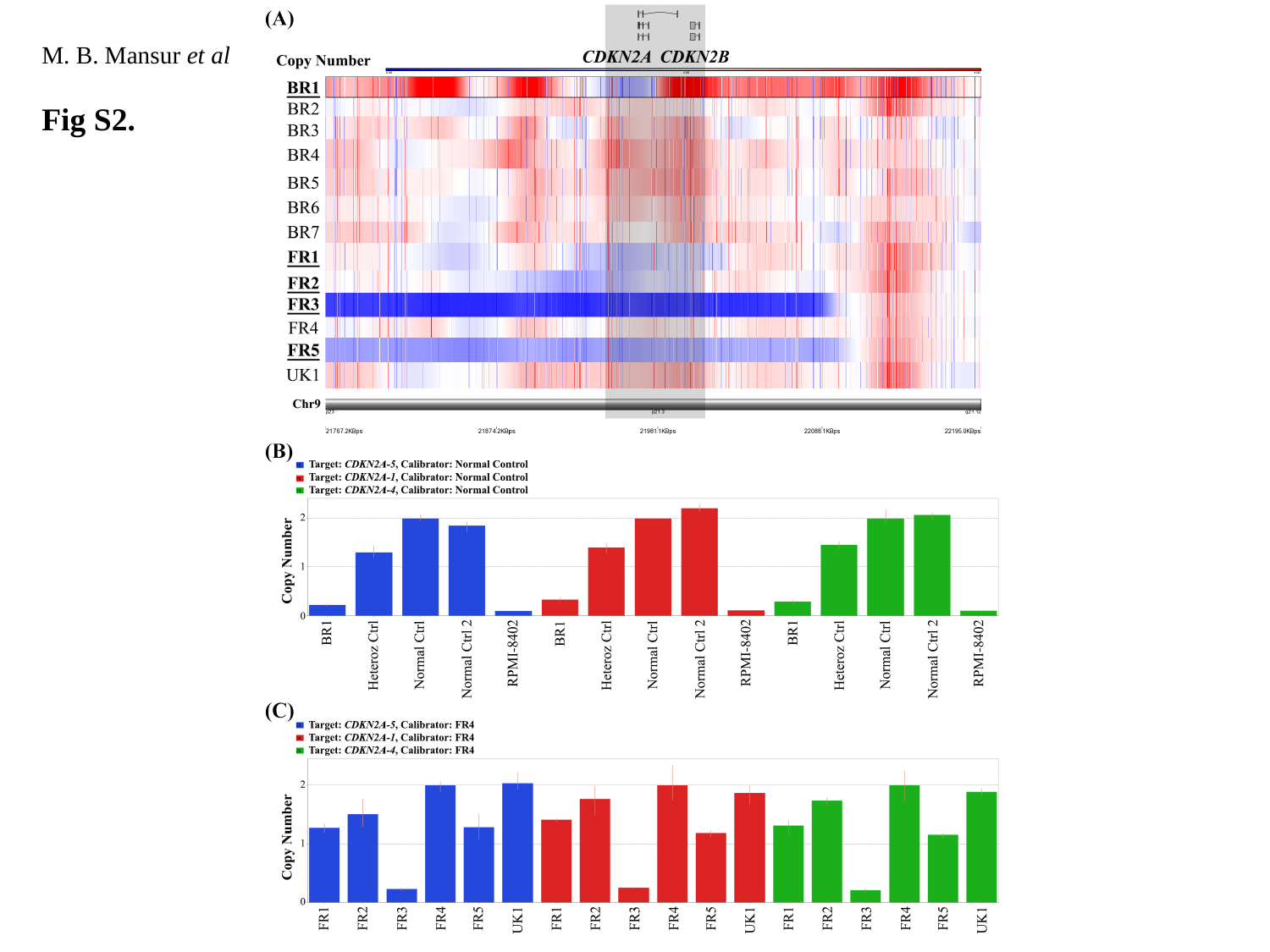

M. B. Mansur et al
Fig S2.

Supplement: Supplementary file 3 — Fig S2. Genomic copy number analysis of RB1. [file BJH-171-574-s003.ppt]

## Slide 1
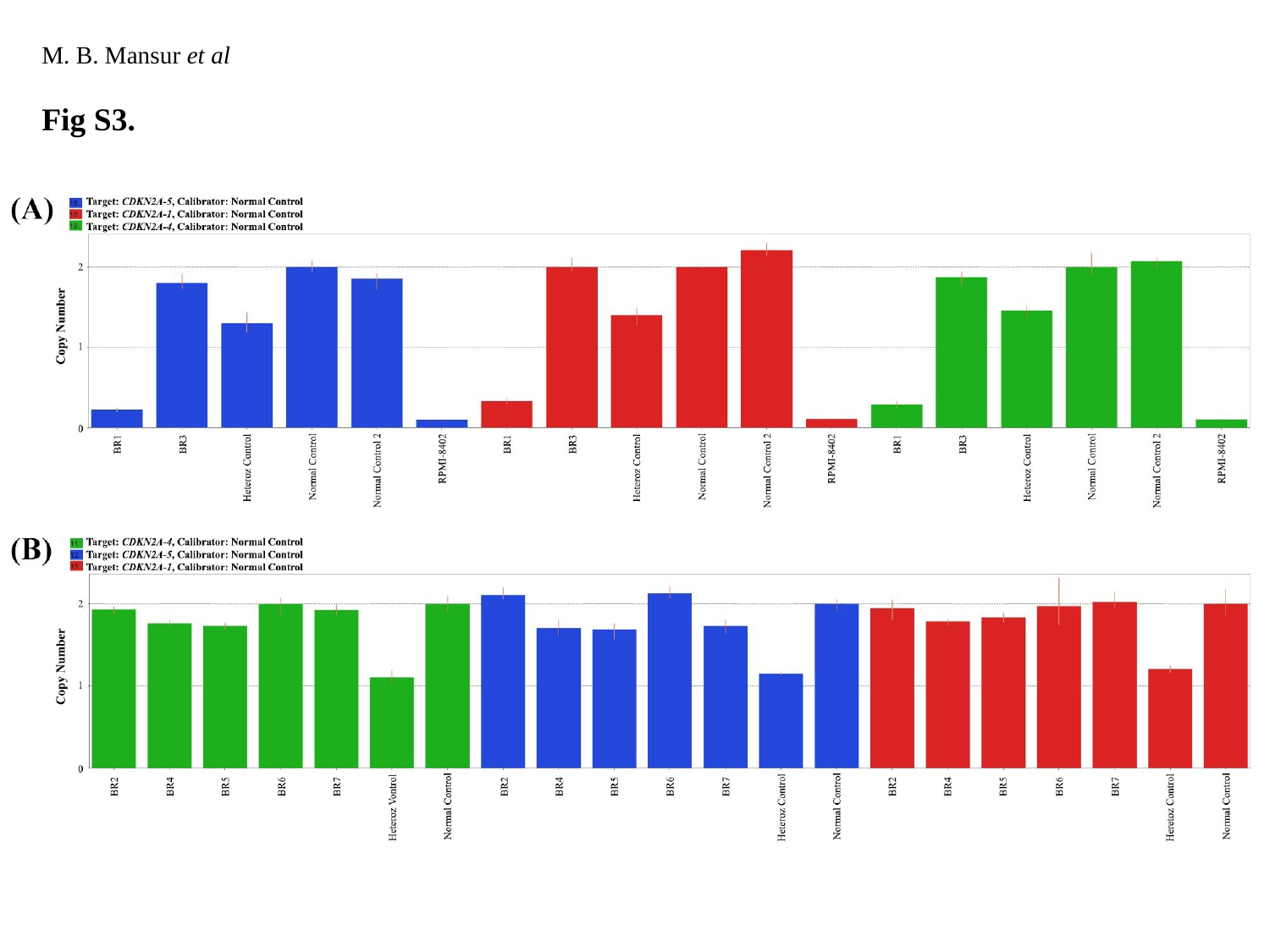

M. B. Mansur et al
Fig S3.

Supplement: Supplementary file 4 — Fig S3. CDKN2A Q‐PCR copy number data of the Brazilian patients. [file BJH-171-574-s004.ppt]

## Slide 1
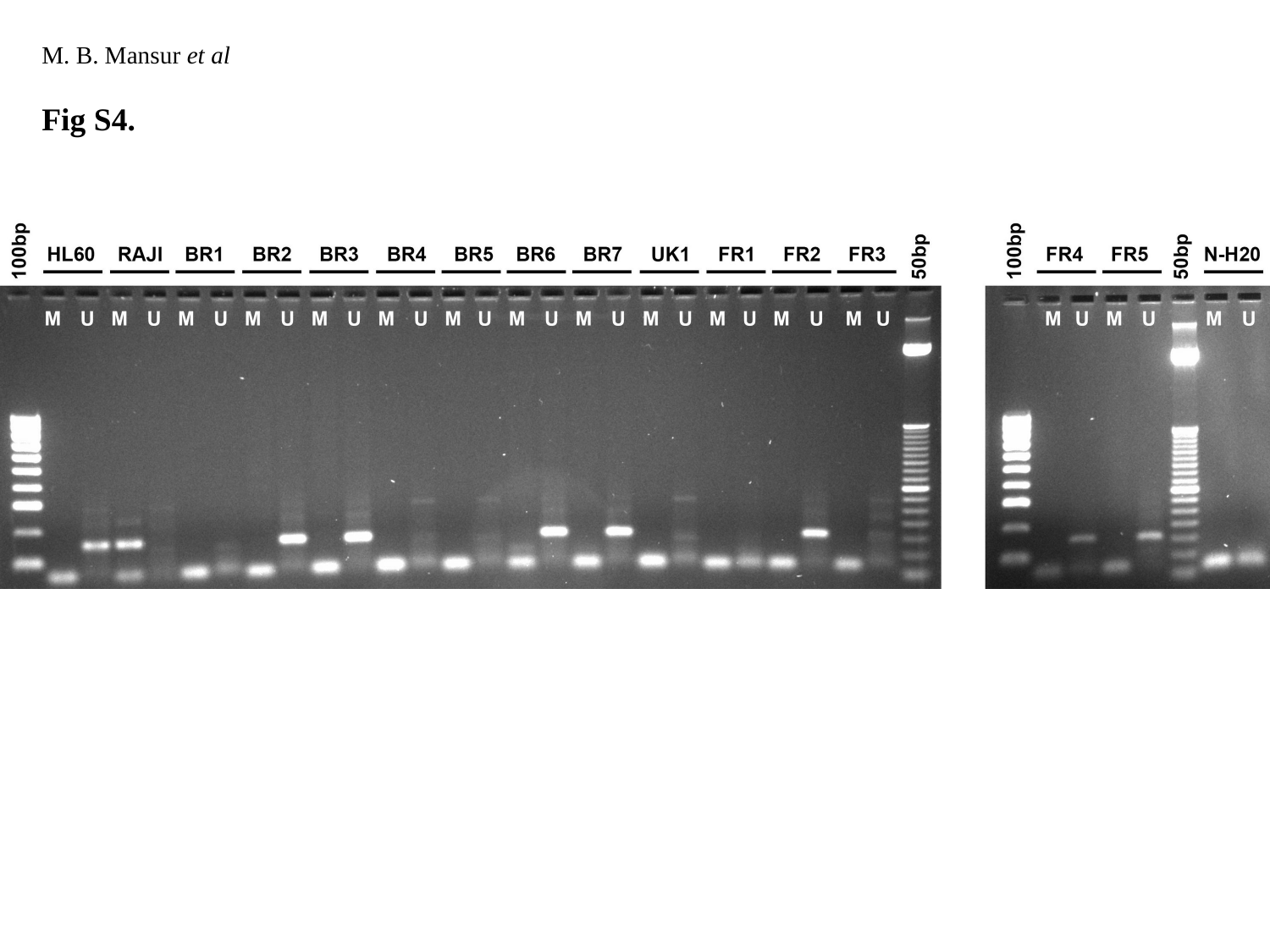

M. B. Mansur et al
Fig S4.

Supplement: Supplementary file 5 — Fig S4. Methylation specific PCR (MSP) in our iT‐ALL cohort and two control cell lines. [file BJH-171-574-s005.ppt]
