# Supplementary material for: Distinctive genotypes in infants with T‐cell acute lymphoblastic leukaemia
Source: Br J Haematol. 2015 Jul 24;171(4):574–84. doi: 10.1111/bjh.13613 (PMC4737125; doi:10.1111/bjh.13613)
Supplement: Supplementary file 6 — Table SI. Clinical–demographic data of infant T‐ALL cases. [file BJH-171-574-s006.doc]

**Table SI. Clinical-demographic data of infant T-ALL cases**

| ***Patient ID*** | ***Age*** | ***Gender*** | ***WBC x10^9^/L (x109/L)*** | ***Blast %*** | ***Mediastinal Mass*** | ***SCT*** | ***Treatment Protocol**** | ***Outcome*** |
| --- | --- | --- | --- | --- | --- | --- | --- | --- |
| ***BR1*** | 12m | Male | 80.0 | 80 | No | Yes | GBTLI-1999 | Deceased |
| ***BR2*** | 8m | Male | 74.1 | 60 | No | Yes | GBTLI-1993 | Deceased |
| ***BR3*** | 6m | Female | 131.6 | 89 | Yes | NA | BFM-1995 | Deceased |
| ***BR4*** | 7m | Female | 53.2 | 78 | No | No | BFM-2002 | Deceased |
| ***BR5*** | 11m | Male | 65.8 | 80 | No | No | INTERFANT-2006 | Deceased |
| ***BR6*** | 7m | Female | 362.6 | 94 | No | Yes | GBTLI-1999 | Alive in CCR |
| ***BR7*** | 8m | Male | 21.6 | 43 | No | No | BFM-2002 | Deceased |
| ***UK1*** | 9m | Male | 236.1 | 80 | No | NA | INTERFANT-2006 | Deceased |
| ***FR1*** | 9m | Female | 165.0 | 82 | Yes | NA | INTERFANT-2006 | Alive in CCR |
| ***FR2*** | 11m | Female | 150.0 | 95 | No | No | INTERFANT-1999 | Deceased |
| ***FR3*** | 12m | Male | 380.0 | 100 | No | No | INTERFANT-2006 | Deceased |
| ***FR4*** | 11m | Female | 60.0 | 74 | No | Yes | EORTC-58951 | Deceased |
| ***FR5*** | 9m | Female | 290.0 | 95 | No | Yes | EORTC-58081 | Alive in CCR |

Abbreviations: ID = identification; m = months; WBC = white blood cells count; CCR = complete continuous remission; SCT = stem cell transplant; NA = data not available. *Brazilian T-ALL patients were treated outside of clinical trials. Thus, all patients were treated according to previously published protocols with risk-adapted strategies for infant ALL (Brandalise, et al 2010, Pieters, et al 2007, Stary, et al 2014).
